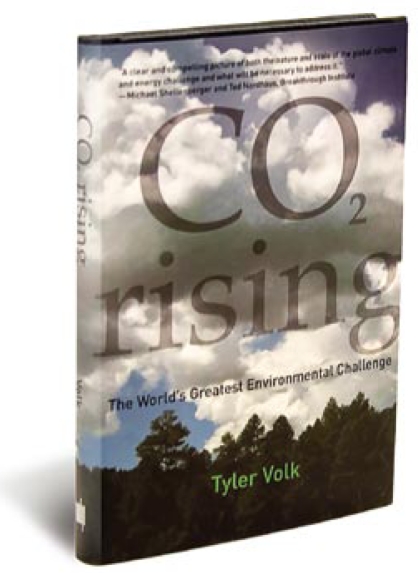# CO_2_ Rising: The World’s Greatest Environmental Challenge

**Published:** 2009-02

**Authors:** Kristie L. Ebi

**Affiliations:** Kristie L. Ebi is an independent consultant who has been conducting research on the impacts of and adaptation to climate change for more than a decade. She was a lead author for the “Human Health” chapter of the IPCC Fourth Assessment Report. She has edited four books on climate change and has more than 80 publications

Avoiding, preparing for, and effectively responding to the significant challenges posed by climate change require extensive engagement across scientific disciplines, decision makers, and the public. The multidisciplinary nature of climate change and the speed with which it has moved from being the concern of a few to affecting the lives of millions mean that few people have received the education or training needed to understand the drivers of Earth’s climate, the current and projected consequences of climate change for the biosphere and all living things, and the large-scale changes in technologies and policies needed to address these risks. *CO_2_* *Rising* is an important addition to a growing literature aimed at providing a basic understanding of key aspects of climate change.

The book focuses on the global carbon cycle, which describes the circulation of carbon atoms throughout the biosphere. Basic knowledge of the carbon cycle is fundamental to understanding why anthropogenic burning of fossil fuels and deforestation are responsible for recent and future increases in global temperatures, changes in the hydrologic cycle, increasing extreme weather events, ocean acidification, and other impacts. The book includes chapters that trace the paths of carbon atoms (anthropogenic and natural) through various pools in the carbon cycle; describe some of the critical evidence demonstrating that the recent worldwide increase in carbon dioxide is anthropogenic in origin; discuss projections of future CO_2_ concentrations under different assumptions of technology change; summarize some technologies that may help reduce emissions of CO_2_; and discuss the geopolitical and social justice issues that are increasingly driving international negotiations on how to address climate change. No more than a basic knowledge of science is needed to understand the information presented, making the book highly accessible besides being very instructive.

In addition, the book is well written and engaging. Volk takes an unusual approach by describing the carbon cycle from the perspective of a carbon atom. The main “character” is a carbon atom named Dave after C. David Keeling, the scientist who dedicated his life to monitoring atmospheric CO_2_ and who discovered its rising concentrations. Coalleen, Oiliver, Methaniel, and Icille join Dave; the first three are from fossil fuels and the last from ice core data. Each has a life history that is both whimsical and solidly based in science. For example, since being released from a limestone cliff 32,000 years ago, Dave has recently gone through all the major carbon pools in the biosphere, including the atmosphere, where Dave passed through an infrared gas analyzer at Mauna Loa Observatory; the oceans, where Dave resided in a marine diatom; and the land, where Dave was incorporated in a rice grain in Bangladesh that was subsequently consumed and exhaled, and was later incorporated in a barley grain that was brewed to make beer that the author drank.

The stories of these individual carbon atoms illustrate the movements of carbon through the biosphere, as well as showing why it is humans who are changing the climate through their past and future fossil fuel emissions and deforestation. It is then possible, by linking emissions with the global economy, to estimate the rates at which CO_2_ will continue to increase over the next 50 years and the consequences of that increase for global temperatures. The assumptions and uncertainties associated with these estimates are clearly presented. The final discussions focus on what can be done to slow and eventually stop increasing anthropogenic emissions—another area where Volk provides clear, concise, and balanced information. The challenges of changing our technology infrastructure are not minimized, nor are the consequences for not doing so. Volk lays out why the United States, with 25% of global emissions and significantly larger per capita use of fossil fuels than any country, must play a leading role in finding technologic and diplomatic solutions to rising CO_2_ concentrations.

Volk clearly and fairly communicates complex and sometimes difficult concepts. *CO_2_* *Rising* provides the basic information about the global carbon cycle that is needed to understand the scope, challenges, and options for dealing with climate change. This understanding should be part of everyone’s scientific literacy.

## Figures and Tables

**Figure:**